# Psychological Distress in Outpatients With Lymphoma During the COVID-19 Pandemic

**DOI:** 10.3389/fonc.2020.01270

**Published:** 2020-07-10

**Authors:** Francesca Romito, Miriam Dellino, Giacomo Loseto, Giuseppina Opinto, Erica Silvestris, Claudia Cormio, Attilio Guarini, Carla Minoia

**Affiliations:** ^1^Psycho-Oncology Unit, IRCCS Istituto Tumori “Giovanni Paolo II”, Bari, Italy; ^2^Gynecology Oncology Unit, IRCCS Istituto Tumori “Giovanni Paolo II”, Bari, Italy; ^3^Hematology Unit, IRCCS Istituto Tumori “Giovanni Paolo II”, Bari, Italy; ^4^Unit of Hematology and Cell Therapy, Laboratory of Hematological Diagnostics and Cell Characterization, Bari, Italy

**Keywords:** COVID-19, lymphoma, cancer, psychological distress, post-traumatic stress symptoms

## Abstract

Cancer patients are a population at high risk of contracting COVID-19 and, also of developing severe complications due to the infection, which is especially true when they are undergoing immunosuppressive treatment. Despite this, they had still to go to hospital to receive chemotherapy during lockdown. In this context, we have evaluated the psychological status of onco-hematological outpatients receiving infusion and not deferrable anti-neoplastic treatment for lymphoproliferative neoplasms, with the aim of both measuring the levels of post-traumatic symptoms, depression, and anxiety during the pandemic and also of investigating the perception of risk of potential nosocomial infection. The Impact of Event Scale-Revised (IES-R) and the Hospital Anxiety and Depression Scale (HADS) were administered to all patients. Moreover, patients were investigated about their worries regarding the impact of COVID-19 on their lives as onco-hematologic patients. Since the 2nd to the 29th April 2020 (during the first phase of the lockdown period in Italy), 77 outpatients were prospectively evaluated. They were diagnosed with non-Hodgkin's lymphoma, classical Hodgkin lymphoma, and Chronic lymphocytic leukemia/Small lymphocytic lymphoma. The mean age was 56.6 (range 22–85). We found that 36% of patients had anxiety (HADS-A), 31% depression (HADS-D), and 43% were above the cut-off for the HADS-General Scale; 36% fulfilled the diagnostic criteria for post-traumatic stress disorder (PTSD). Women and younger patients were found to be more vulnerable to anxiety and PTSD. The study firstly analyzes the psychological impact of the COVID-19 pandemic on the frail population of patients affected by lymphoproliferative neoplasms, to underly the importance of screening patients for emotional and distress conditions and then offering them psychological support.

## Introduction

Coronavirus Disease 2019 (COVID-19), the disease caused by SARS-CoV-2, was firstly diagnosed in Wuhan, China, in late October 2019, and rapidly spread all over the world, causing a severe pandemic^1^. In Italy, the first patient was identified on the 18th February, and since that time to the 24th April 192,994 positive cases were identified and 25,969 deaths occurred. The majority of contagions and deaths were observed in the northern part of the Country (Lombardia 55.8%, Emilia Romagna 13.8%, Piemonte 7.5%, Veneto 5.2%), but every region was affected, with the virus particularly affecting large cities.

On 10th March, lockdown measures were imposed on the Italian population with the aim of limiting viral diffusion and better protecting frail and elderly people (1, 2). The psychological effects of the COVID-19 quarantine on the general population in China showed a high prevalence of symptoms of post-traumatic stress. In particular, intrusive symptoms, states of hyper-vigilance, and negative alterations of mood level were recognized (3, 4). Similarly, in Italy, a survey 18,147 people during the epidemic peak found the presence of high levels of post-traumatic stress disorder, depression, anxiety, insomnia, and perceived stress (5). Being of a younger age and female were factors constantly associated with an increased risk of developing major mental health disorders (5). The mean age of infected people in Italy was 79 years, with a slight prevalence of males (63.3%), and with more than 80% of cases presenting with two or more comorbidities^2^.

A study on Chinese cancer patients documented a higher risk for this subset of the population of contracting COVID-19 during immune suppression due to anti-neoplastic treatments and, moreover, a higher risk of severe complications leading to the need for intensive care. For this population, there was also a higher risk of death, especially for elderly patients (6–8). In this perspective, there was an urgent need to draw up specific recommendations in order to protect cancer patients and, at the same time, to guarantee continuation of care. Even if the epidemiological data on the incidence of COVID-19 infection in cancer patients and its rate of morbidity and mortality in Italy had not yet been published, since the beginning of March, the Società Italiana di Ematologia (SIE), the Gruppo Italiano Trapianti di Midollo Osseo (GITMO), Fondazione Italiana Linfomi (FIL), and the Associazione Italiana di Oncologia Medica (AIOM) disclosed recommendations on the management of onco-hematologic patients^3^^,^^4^^,^^5^.

Lymphoproliferative neoplasms, including non-Hodgkin's lymphomas (NHL), classical Hodgkin lymphoma (cHL), and Chronic lymphocytic leukemia/Small lymphocytic lymphoma (CLL/SLL), because of better diagnostic tools and targeted therapies, now show a cumulative cure rate of about 70%; one of their main complications, however, is that of opportunistic infections due to the intrinsic and iatrogenic suppression of the immune system (9, 10). As yet, there have been very few studies analyzing the impact of COVID-19 on the outcome of lymphoproliferative neoplasms (11, 12), but the efforts of scientific organizations have been directed at guiding onco-hematologists to a correct management of both inpatients and outpatients, to avoid, as far as possible, contracting the infection and to guarantee the course of treatment. In this first phase, following national recommendations, the majority of non-urgent follow-up visits were postponed, and the majority of oral anti-neoplastic therapies were managed through telephone and email contact, with the patient remaining at home. Furthermore, recommendations suggested to evaluate situations case by case, so as to determine the advantage of administering a potentially immunosuppressive treatment, taking into account age, aggressiveness of the disease, and intention to treat. It was also recommended that outpatients admitted to hospital to receive infusion anti-cancer treatment should undergo a pre-triage by telephone and an in-hospital triage for COVID-19 symptoms (13). Despite these measures, the risk of contracting the infection was always possible, even in a non-COVID cancer institute (14).

It is understandable that this new situation could lead to additional psychological distress during the treatment period, due to fears surrounding delaying treatments and invalidating prognoses, and of contracting the infection inside the hospital. It also has to be considered that the presence of a caregiver inside the hospital was not allowed for patients not in need of assistance. It is already well-recognized that cancer patients are at an increased risk of experiencing psychological distress with respect to the general population (15, 16), as cancer is a concrete threat to the life of the person and may deeply modify her/his social, emotional, and relational world. Psychological distress and post-traumatic stress symptoms in cancer patients during the COVID-19 pandemic have not yet been evaluated in Chinese or other national studies. In this context, we conducted a prospective evaluation of the psychological status of outpatients receiving anti-neoplastic treatment for lymphoproliferative diseases during lockdown in our non-COVID Cancer Center Institute in southern Italy, pursuing the following aims: (i) to measure the levels of post-traumatic symptoms, depression, and anxiety during the pandemic; and (ii) to investigate the perception of the risk of potential nosocomial infection.

## Materials and Methods

### Study Design and Psychological Distress Evaluation

Consecutive outpatients diagnosed with a lymphoproliferative neoplasm were prospectively enrolled in the study as they arrived in our clinic to receive infusion chemo- or immunotherapy. Inclusion criteria were: age ≥18 years; diagnosis of lymphoproliferative neoplasm, including cHL, NHL, or CLL/SLL; intravenous chemotherapy or immunotherapy (induction phase, second line, more than 3 lines, maintenance therapy); and having signed the informed consent to participate in the study. Patients enrolled in clinical trials were also included. Patients receiving oral therapy for their lymphoma or CLL were excluded. All data were collected from the 2nd April to the 29th April 2020, during strict lockdown measures in Italy.

Approval for the study was obtained from the local ethical committee.

The Impact of Event Scale-Revised (IES-R) (17) and the Hospital Anxiety and Depression Scale (HADS) (18) were administered to all patients.

The IES-R is a self-administered questionnaire measuring a person's subjective reaction after a traumatic event, leading to the diagnosis of PTSD. The IES-R is composed of 22 items divided into three subscales measuring avoidance, intrusion, and hyperarousal. Answers range on a scale from 0 (not at all) to 4 (extremely) (17).

The HADS is a self-administered questionnaire developed to detect the state of anxiety and depression in non-psychiatric patients with organic disease. The HADS is composed of 14 items, seven of which measure anxiety (HADS-Anxiety, HADS-A) and the other seven measuring depression (HADS-Depression, HADS-D) on a four point Likert scale (18). The scale has demonstrated satisfactory psychometric characteristics in cancer patients and has been translated and validated in Italian populations (19, 20). Both questionnaires referred to the last 7 days.

Patients also answered a brief structured interview, investigating their worries regarding the impact of COVID-19 on their lives as onco-hematologic patients, and their need for psychological help (21). The following questions were asked: (1) What is your largest concern in this period? (a) the risk of delaying the chemotherapy administration due to COVID-19, (b) the risk of getting infected while in hospital, (c) the risk of infecting my relatives coming back home, (d) potential difficulties in contacting my onco-hematologist in the case of need, (e) social distancing from my loved ones, or (f) financial difficulties. (2) Have your worries increased during the pandemic? (Answer: Yes; No). (3) Did you feel the need for: (a) psychological support; (b) homeopathic/herbal remedies; (c) drugs for anxiety, insomnia, depression; (d) other; or (e) no help. (4) Do you need online psychological support from the Psycho-Oncology Unit of the Hospital? (Answer: Yes; No).

### Data Collection and Statistical Analysis

Data were collected in a dedicated database. A descriptive analysis was performed for sample description and frequencies and for IES-R and HADS subscales results (HADS-A, HADS-D, and HADS-General). A correlation analysis was implemented to detect the univariate associations between sociodemographic characteristics, clinical data, and the IES-R score, as well as the subscales of the HADS. The correlation between the continuous variables was calculated using Pearson's Correlation Coefficient (r). Eta squared was used for comparison between categorical variables and continuous variables. Student *t*-test was performed to assess the differenc**e** between the mean value in two groups. Statistical significance was achieved at a *p* < 0.05. All statistical analyses were performed using the using the R statistical environment, version 3.5.2 (The R Foundation for Statistical Computing; Vienna, Austria).

## Results

### Patients' Clinical and Sociodemographic Characteristics

Seventy-seven outpatients were enrolled at the Hematology Unit of the IRCCS “Giovanni Paolo II” in Bari. The mean age was 56.6 (range 22–85); 39 (50.6 %) were male and 38 (49.4 %) female. Diagnoses were distributed as: cHL n. 25 (32.5%); aggressive NHLS n. 15 (19.5%), of whom n. 9 had diffuse large B-cell lymphoma (DLBCL), n 5 mantle cell lymphoma (MCL), and n. 1 primary mediastinal large B-cell lymphoma; indolent NHLs n. 37 (48%), distributed as follicular lymphoma (FL) n. 28, marginal zone lymphoma (MZL) n. 3, hairy cell leukemia (HCL) n. 3, lymphoplasmacytic lymphoma n. 1, CLL/SLL n. 3. N. 6 (7.8%) were treated inside of a clinical trial. N. 52 (67.5%) of patients received infusion chemotherapy or immune-chemotherapy and n. 25 (32.5%) received immunotherapy. According to the line of treatment, n. 38 (49.3%) received induction therapy, n. 6 (7.8%) second line therapy, n. 13 (16.9%) were at the third or higher line, and n. 20 (26%) were receiving maintenance therapy.

Considering the sociodemographic characteristics, 34 (44%) had a low level of education (up to 8 years), 23 (30%) a medium level (up to 13 years), and 17 (22%) a high level of education. Six patients (8%) lived alone, 47 (61%) with one or two family members, and 21 (27%) with three or more family members. Concerning their working life, 42 (54%) were not working (namely housewives, retired, unemployed, or students) and 32 (42%) were occupied. Data were missing for three patients in regards to working life.

Patients' clinical and sociodemographic characteristics, along with chemotherapeutic regimens, are described in Table 1.

**Table 1:** Patients' clinic and sociodemographic characteristics.

**Patients' characteristics**	***N* = 77**	**% (100)**
Age at evaluation, years (mean, range)	56.6 (22–85)	
Male	39	50.6
Female	38	49.4
Histotype		
Classical Hodgkin lymphoma	25	32.5
Aggressive NHLs	15	19.5
Diffuse large B-cell lymphoma	9	11.7
Primary mediastinal large B-cell lymphoma	1	1.3
Mantle cell lymphoma	5	6.5
Indolent NHLs	37	48
Follicular lymphoma	28	36.4
Marginal zone lymphoma	3	3.9
Hairy cell leukemia	3	3.9
Lymphoplasmacytic lymphoma	3	3.9
Chronic lymphocytic leukemia /small	1	1.3
Lymphocytic lymphoma	3	3.9
Line of chemotherapy		
1st	38	49.3
2nd	6	7.8
>/= 3rd	13	16.9
Maintenance	20	26
Clinical trial		
Yes	6	7.8
No	94	92.2
Type of therapy		
Chemotherapy or immunochemotherapy	52	67.5
Immunotherapy	25	32.5
Type of chemotherapeutic regimen		
ABVD	15	19.5
Brentuximab vedotin	7	9
BeGEV	1	1.3
Nivolumab	2	2.6
R-CHOP/R-COMP	8	10.4
R-bendamustine	12	15.6
R-DHAP/R-DHAOx	3	3.9
Gemcitabine-navelbine	2	2.2
Pentostatine	3	3.9
FCR	1	1.3
Rituximab	18	23.4
Obinutuzumab	5	6.5
Education (years of schooling)		
>8 years	34	44%
9–13	23	30%
14–18	17	22%
Missing	3	4%
Number of cohabitants		
Living alone	6	8%
1–2	47	61%
>3	21	27%
Missing	3	4%
Job		
Not working (housewife, retired, unoccupied, student)	42	54%
Working	32	42%
Missing	3	4%

### Levels of the IES-R and HADS

The mean IES-R score of patients was 19.7 (SD ± 13.9), with 64% (n. 49) not showing a PTSD diagnosis (score <23) and 36% (n. 28) indicating the fulfillment of diagnostic criteria for PTSD (score ≥24) at a mild (score 24–32), moderate (score 33–36), or severe level (score >37). Mean scores for the IES-R subscales were: avoidance 7.55 (SD ± 5.50), intrusion 6.22 (SD ± 5.06), and hyperarousal 5.92 (SD ± 4.98).

The mean HADS-General Scale score (HADS-GEN) was 12.1 (SD ± 6.4). We found that 43% of patients (n. 33) were above the cut-off (score ≥13) for the general scale, 36% (n. 28) were above the cut-off (score ≥8) for HADS-A (HADS-Anxiety cases), and 31% (n. 24) for HADS-D (HADS-Depression cases) (Table 2).

**Table 2:** Analysis of anxiety, depression and PTSD.

		***N* (77)**	**% (100)**
HADS-GEN	M 12,15 (6,48 SD)		
	No case	44	57
	Case (≥ 13)	33	43
HADS-A	M 6,22 (3,94 SD)		
	No case	49	64
	Case (≥ 13)	28	36
HADS-D	M 5,93 (3,44 SD)		
	No case	53	69
	Case (≥ 13)	24	31
IES-R	M 19,7 (13,92 SD)		
	No diagnosis of PTSD (23 or below)	49	64
	PTSD mild (24–32)	14	18
	PTSD moderate (33–36)	6	8
	PTSD severe (>37)	8	10
IES-R Intrusion	M 6,22 (5,06 SD)		
IES-R Avoidance	M 7,55 (5,50 SD)		
IES-R Hyperarousal	M 5,92 (4,98 SD)		

When the age groups (18–50; 50–70 and >70) were correlated with the dependent variables, higher levels of PTSD (IES-R) were found in the younger age group (namely 18–50) (*r* = 0.43, *p* = 0.03). Moreover, female patients presented with higher levels at the HADS-A (*p* = 0.03) and at the IES-R (*p* = 0.0001) compared with males. No correlations were documented for the other study variables, namely aggressiveness of the disease (aggressive NHL vs. indolent NHL vs. cHL), type of therapy (immunotherapy vs. chemotherapy), line of therapy (1st-2nd-3rd vs. maintenance), or clinical trial vs. standard practice.

The HADS-D has been found to be significantly correlated with IES-R (*r* = 0.40; *p* = 0.0002) and with all the subscales of the IES-R, namely avoidance (*p* = 0.003), intrusion (*p* = 0.003), and hyperarousal (*p* = 0.0001).

We aimed to verify whether levels of HADS-A, HADS-D, HADS-GEN, and IES-R showed differences as time progressed during lockdown. In particular, we have looked for differences between weeks 1–2 and 3–5 of the study corresponding to the peak of contagions in our region, but no differences over the weeks emerged by Student *t*-test.

### Worries Questions

Seventy-five percentage of patients (n. 58) stated that their worries had increased during the pandemic; their most important concerns were: (i) the risk of getting infected while at hospital (32.46%, n. 25), ii) the risk of delaying therapy (20.77%, n. 16), (iii) the risk of infecting relatives coming back home (18.18%, n. 14), (iv) social distancing from their loved ones (12.98%, n. 10), (v) financial difficulties (5.14%, n. 4), and (vi) potential difficulties in contacting the onco-hematologist in case of need (3.89%, n. 3).

They felt the need for psychological support (6%; n. 5), homeopathic or herbal remedies (3%, n. 2), psychotropic drugs (3%, n. 2), or other kinds of unspecified support (13%, n. 10); but in the face of heightened need, the majority sought no kind of help (75%, n. 58).

The request for psychological online support was expressed by 25% (n. 19) of patients. Patients who expressed this need have been contacted by a psychotherapist for online sessions offering psychological support (Table 3).

**Table 3:** Worries regarding the impact of COVID-19 as hematologic patients.

	***N* (77)**	**% 100**
**Are your worries increased during the pandemic?**		
Yes	58	75
No	19	25
**What is your bigger concern in this pandemic?**		
The risk of delaying therapy	16	20.77
The risk of getting infected while at Hospital	25	32.46
The risk of infecting relatives	14	18.18
Potential difficulties in contacting my onco-hematologist	3	3.98
The social distancing from my loved ones	10	12.98
Financial difficulties	4	5.14
**Did you feel the need for:**		
No need of help	58	75
Psychological support	5	6
Homeopathic/herbal remedies	2	3
Drugs for anxiety, insomnia, depression	2	3
Other	10	13
**Do you need an on-line psychological support?**		
Yes	19	25
No	58	75

## Discussion

At the end of February, Italy was severely hit by the COVID-19 pandemic. The significant lockdown measures primarily involved hospitals, both for hospitalized and outpatients. The Apulia region, where this study was conducted, ranks as a region at an intermediate incidence level, in the middle of northern Italy and other regions of central and southern Italy (21). By 22nd April 2020 3,730 cases had been reported in Apulia (median age 58 years old), of whom 624 were hospitalized^6^. The registered deaths were 9.7% of the reported cases. On the 23rd April, a regional incidence of 95.31 cases/100,000 inhabitants was reported^7^.

In the present study, from the 2nd to 29th April, we analyzed the psychological status of outpatients coming to our non-COVID Cancer Institute to receive infusion and not deferrable chemo- or immunotherapy for their lymphoproliferative neoplasm. These patients underwent validated questionnaires measuring anxiety, depression, and PTSD. Data from the evaluation of 77 patients documented that 36% presented with anxiety (HADS-A), 31% depression (HADS-D), and 43% were above the cut-off for the HADS-General Scale. Meanwhile, 36% fulfilled the diagnostic criteria for PTSD. Women have been found to be more vulnerable to anxiety and PTSD and this datum confirms previous literature (22). Younger patients showed higher levels of PTSD. Moreover, the HADS-D subscale was found correlated with the IES-R scores.

As regards PTSD, our results are comparable to other vulnerable populations analyzed during the pandemic. In particular, one of the available cohorts includes psychiatric (non-psychotic) Chinese patients, who reported higher levels of PTSD (mean 17.7; SD ± 13,9) compared to the general Chinese population (mean 11. 3; SD ± 10,1) (23).

Also considering anxiety and depression, no data on cancer patients during the COVID-19 pandemic are available yet. However, if we compare the present cohort with historical cohorts of patients diagnosed with lymphoproliferative neoplasms, we can state that levels of HADS found in our study are significantly higher on both the depression subscale and on the general scale (24).

These findings may be explained considering that the COVID-19 pandemic represents a new form of stressor or trauma (25), that would particularly affect people who are already vulnerable due to other biological or psychological burdens, in this case cancer (26). Moreover, the social isolation imposed during quarantine can increase loneliness and limit social interactions, well-known risk factors for psychopathological problems, including depression (26). These stressors are to be expected as factors in increasing the pre-existing burden of carrying a cancer diagnosis.

In parallel with quantitative analysis, a qualitative evaluation was conducted, with the aim to understand patients' worries when they came in to contact with the hospital environment. The majority of patients (75%) stated that their worries had increased during the pandemic. Their principal worries were in contracting the virus during their stay in hospital (32.46%), delaying chemotherapy (20.77%), and infecting their relatives (18.8%). Following this evaluation, 25% of patients asked to be and were contacted for online sessions of psychological support by a psychotherapist from the Clinics' Unit of Psycho-Oncology. This aspect is particularly interesting since the lockdown massively disrupted the availability of usual face-to-face mental health services, with the option of online support more difficult both to propose and to access.

Some limitations of the study should be discussed. Firstly, the absence of a baseline (pre-COVID-19 pandemic) assessment of enrolled patients means that we cannot ascertain whether the distress reactions have been elicited by the diagnosis or by the pandemic and could thus be considered the exacerbation of a pre-existing mental health problem. The second limitation is that the accrual was conducted in a single institution.

Despite these limitations, to our knowledge, this is the first study that focuses on the psychological aspects of onco-hematologic patients during the COVID-19 pandemic, showing that these patients are at risk of displaying high levels of symptoms of PTSD, anxiety, and depression. The European Psychiatric Association states that one of the consequences of the pandemic could be that psychological issues are considered less important than physical ones, leading to an underestimation of the problem and then to a subsequent increase of psychiatric need in the coming weeks or months (26). This aspect should lead clinicians to reconsider the current practices (26). Given these considerations, we underline the importance of paying considerable attention to the psychological needs of onco-hematologic patients during this upsetting period, by scheduling routine psychological screening of their emotional and stress conditions.

## Data Availability Statement

The raw data supporting the conclusions of this article will be made available by the authors, without undue reservation.

## Ethics Statement

The studies involving human participants were reviewed and approved by Ethical Committee of the IRCCS Istituto Tumori Giovanni Paolo II - Bari, Italy. The patients/participants provided their written informed consent to participate in this study.

## Author Contributions

FR, CM, and MD conceptualized the study. GL, CM, ES, and AG conduced the clinical enrollment. FR and CC conducted the psychological evaluations. GO performed the statistical analysis. All authors contributed to the article and approved the submitted version.

## Conflict of Interest

The authors declare that the research was conducted in the absence of any commercial or financial relationships that could be construed as a potential conflict of interest.

## Footnotes

^1^https://www.who.int/emergencies/diseases/novel-coronavirus-2019

^2^http://www.salute.gov.it/portale/nuovocoronavirus/dettaglioContenutiNuovoCoronavirus.jsp?area=nuovoCoronavirus&id=5351&lingua=italiano&menu=vuoto

^3^http://www.siematologia.it/files/COVID19-Raccomandazioni-SIE-GITMO.pdf

^4^https://ricercatori.filinf.it/wp-content/uploads/2020/03/circolare-minsal-onco.pdf.pdf.pdf

^5^https://www.aiom.it/wp-content/uploads/2020/03/20200313_COVID-19_indicazioni_AIOM-CIPOMO-COMU.pdf

^6^https://www.regione.puglia.it/web/press-regione/-/bollettino-epidemiologico-covid-22.4.2020

^7^https://www.epicentro.iss.it/coronavirus/bollettino/Bollettino-sorveglianza-integrata-COVID-19_23-aprile-2020.pdf
